# Breast Atypical Apocrine Adenosis: A Case Report and Literature Review

**DOI:** 10.7759/cureus.8624

**Published:** 2020-06-15

**Authors:** Kashuf A Khan, Firas Alkistawi, Philip Idaewor, Marina Barron, Abdalla Saad Abdalla Al-Zawi

**Affiliations:** 1 General Surgery, Royal Shrewsbury and Telford Hospital NHS Trust, Shrewsbury, GBR; 2 Surgery, Basildon and Thurrock University Hospital, Basildon, GBR; 3 General Surgery, Basildon and Thurrock University Hospital, Basildon, GBR; 4 General Surgery, Royal Victoria Hospital, Belfast, GBR; 5 General Surgery, Mid and North Essex University Hospital Group, Basildon, GBR; 6 Breast Surgery, Basildon and Thurrock University Hospital, Basildon, GBR; 7 Breast Surgery, Anglia Ruskin University, Chelmsford, GBR

**Keywords:** atypical apocrine adenosis, sclerosing adenosis, breast disease, lobular carcinoma in situ, apocrine ductal carcinoma in situ

## Abstract

Atypical apocrine adenosis (AAA) is a benign lesion of the breast that is identified more frequently today than in the past when it was considered a rare diagnosis and commonly misdiagnosed as other malignant lesions of the breast. AAA is defined as the presence of apocrine cytology in a recognisable lobular unit associated with sclerosing adenosis. We present a case of an incidental finding of AAA and discuss diagnostic challenges and their implications on clinical management.

## Introduction

Some breast lesions are associated with apocrine phenotype features, such as atypical apocrine adenosis (AAA), apocrine ductal carcinoma in situ (DCIS), and invasive carcinoma with apocrine features [1].

AAA is a rare benign breast lesion and should not be regarded as a direct histologic precursor to invasive breast carcinoma. The presence of significant cytological atypical changes in the breast apocrine cells (e.g., nuclear enlargement, prominent/multiple nucleoli, and hyperchromasia) is known as apocrine atypia. Breast AAA is defined as the presence of apocrine cytology in a recognisable breast lobular unit associated with sclerosing adenosis [2].

## Case presentation

**Figure 1:**
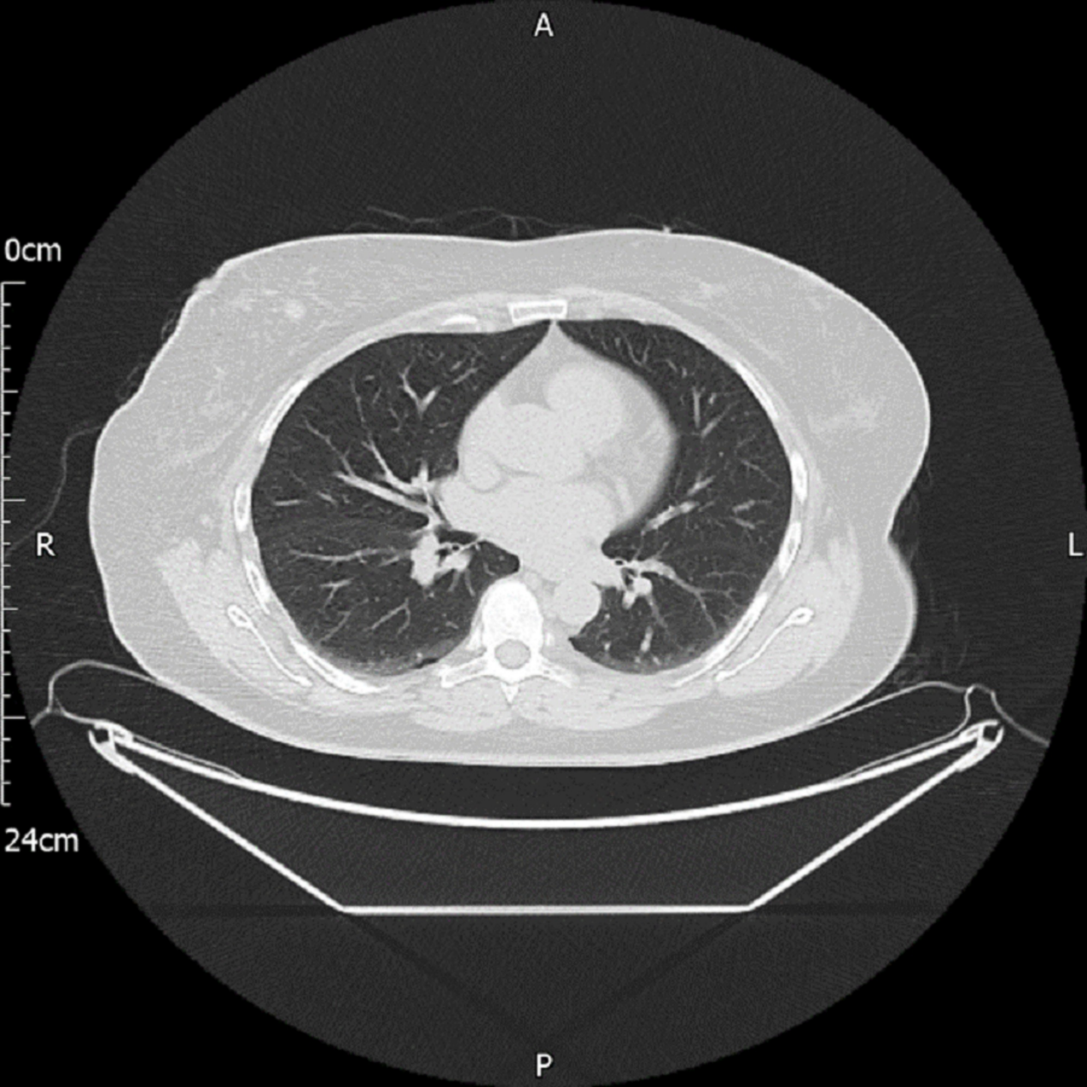
Chest computed tomography showing a small lesion in the right breast

A 54-year-old woman underwent coronary angiogram computed tomography (CT) for chest pain. The CT revealed only an incidental right breast small nodule (Figure 1). Her past medical history and family history were unremarkable. The findings of her breast examination were also unremarkable.

**Figure 2:**
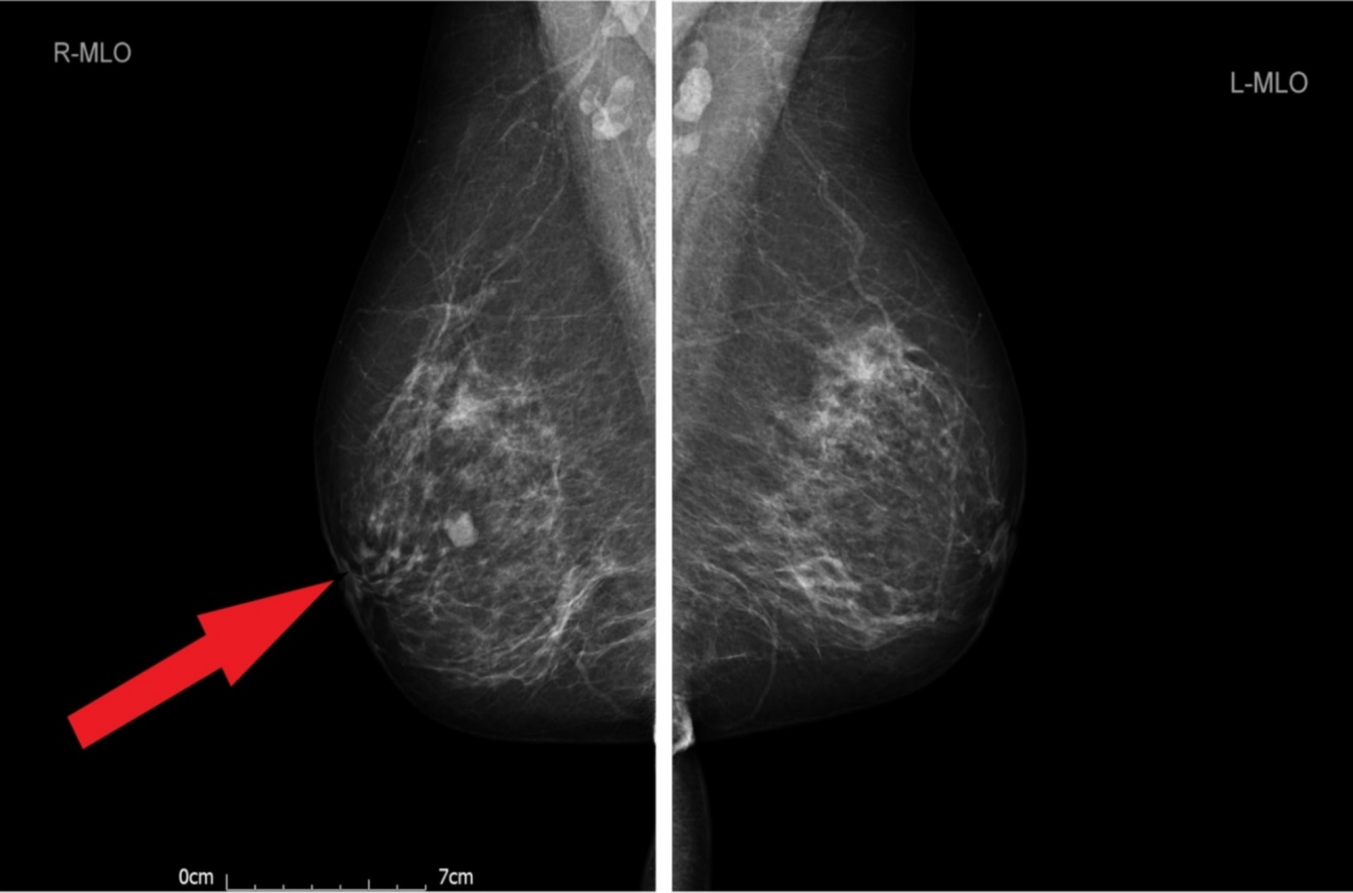
Mammogram showing the right breast lesion

**Figure 3:**
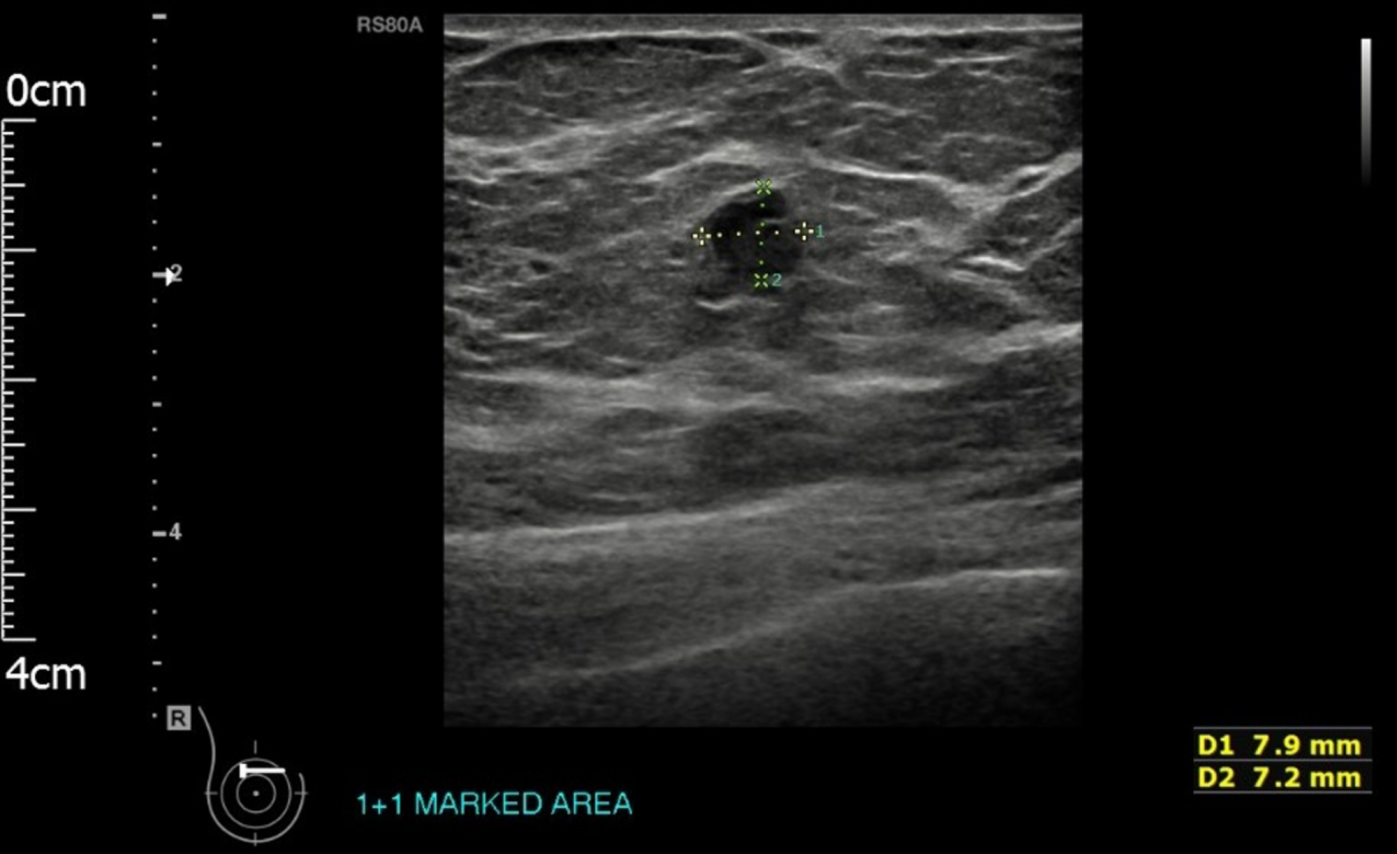
Right breast ultrasound showing the right breast lesion

Her mammogram (Figure 2) revealed a well-defined soft tissue nodule measuring 11 mm in the medial part of the right breast (Breast Imaging, Reporting, and Data System [BI-RADS] grade M2). Right breast ultrasonography (Figure 3) showed a lobulated hypoechoic lesion with slightly ill-defined margins in places. Appearances were indeterminate (BI-RADS grade U3).

**Figure 4:**
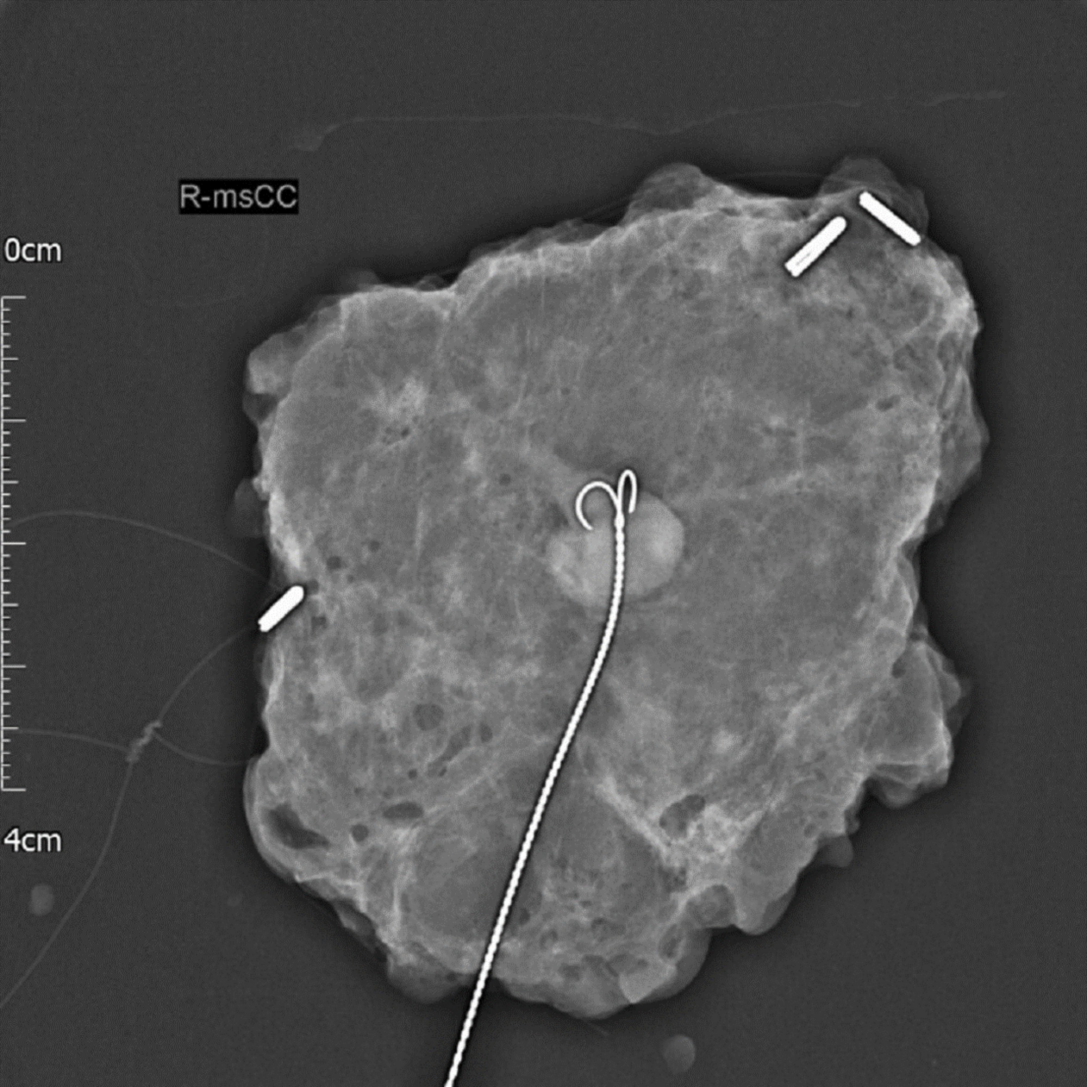
Wire-guided excision biopsy of the lesion

**Figure 5:**
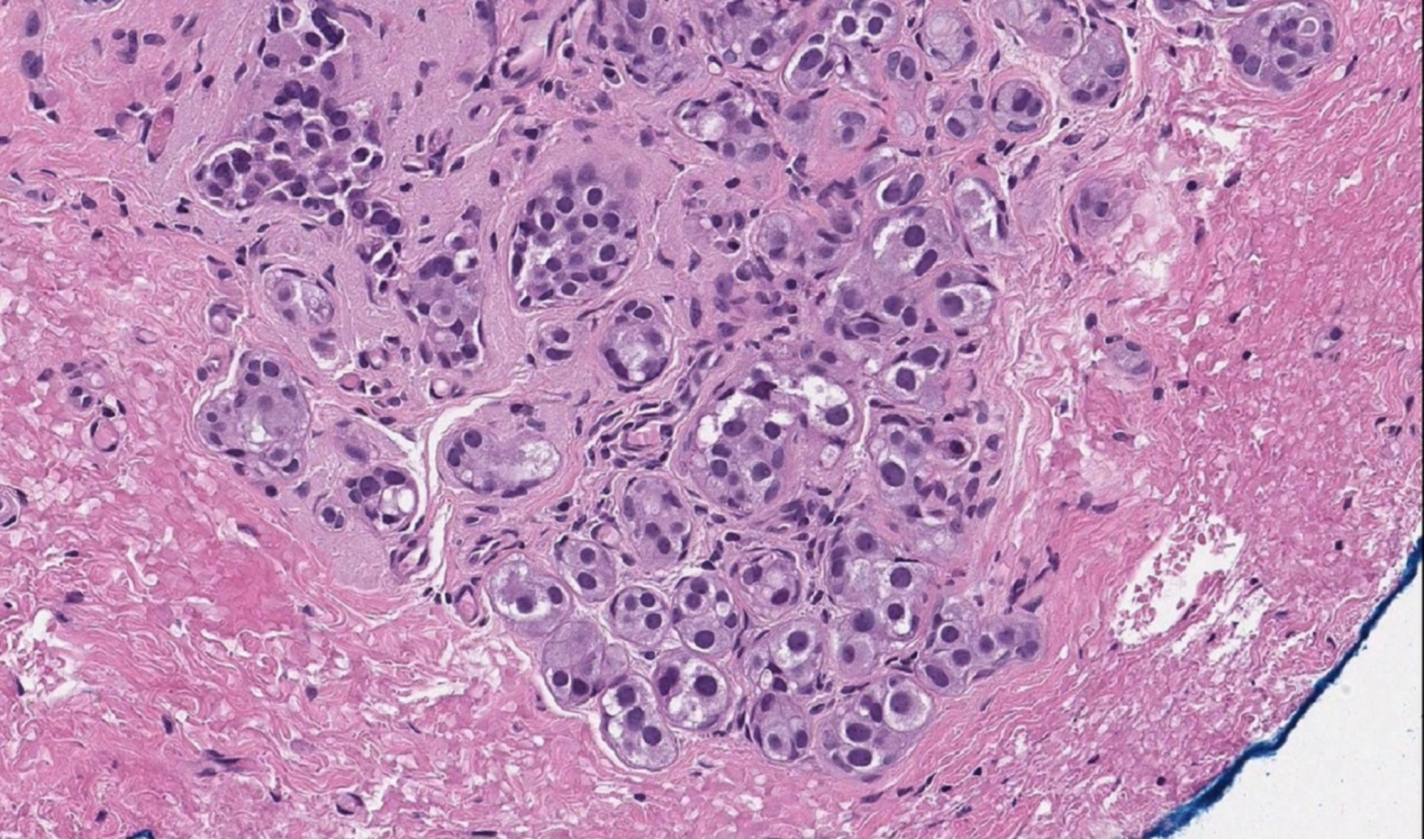
Histopathologic picture of the lesion demonstrating atypical apocrine adenosis. The epithelium is of apocrine type with nuclear pleomorphism in closely packed acini

Core biopsy of breast tissue showed tightly packed acini with the epithelium of apocrine type exhibiting nuclear pleomorphism. The lesional cells were large with abundant eosinophilic cytoplasm and large vesicular nuclei, with prominent eosinophilic nucleoli and formed nests and irregular ducts within a fibrous stroma. The specimen had focal cribriform and solid architecture. Immunohistochemistry studies revealed that the cells are strongly and diffusely positive for gross cystic disease fluid protein (GCDFP)-15 and negative for oestrogen receptors. With p63 and smooth muscle actin (SMA) stains, the myoepithelial cells appeared mostly preserved with a degree of loss in the solid areas. These features were consistent with AAA. The lump was removed surgically with wire-guided localisation (Figure 4), and the postoperative histology confirmed completely excised AAA (Figure 5). After surgery, the patient was advised to continue with the Breast National Screening Programme.

## Discussion

Exocrine glands

**Figure 6:**
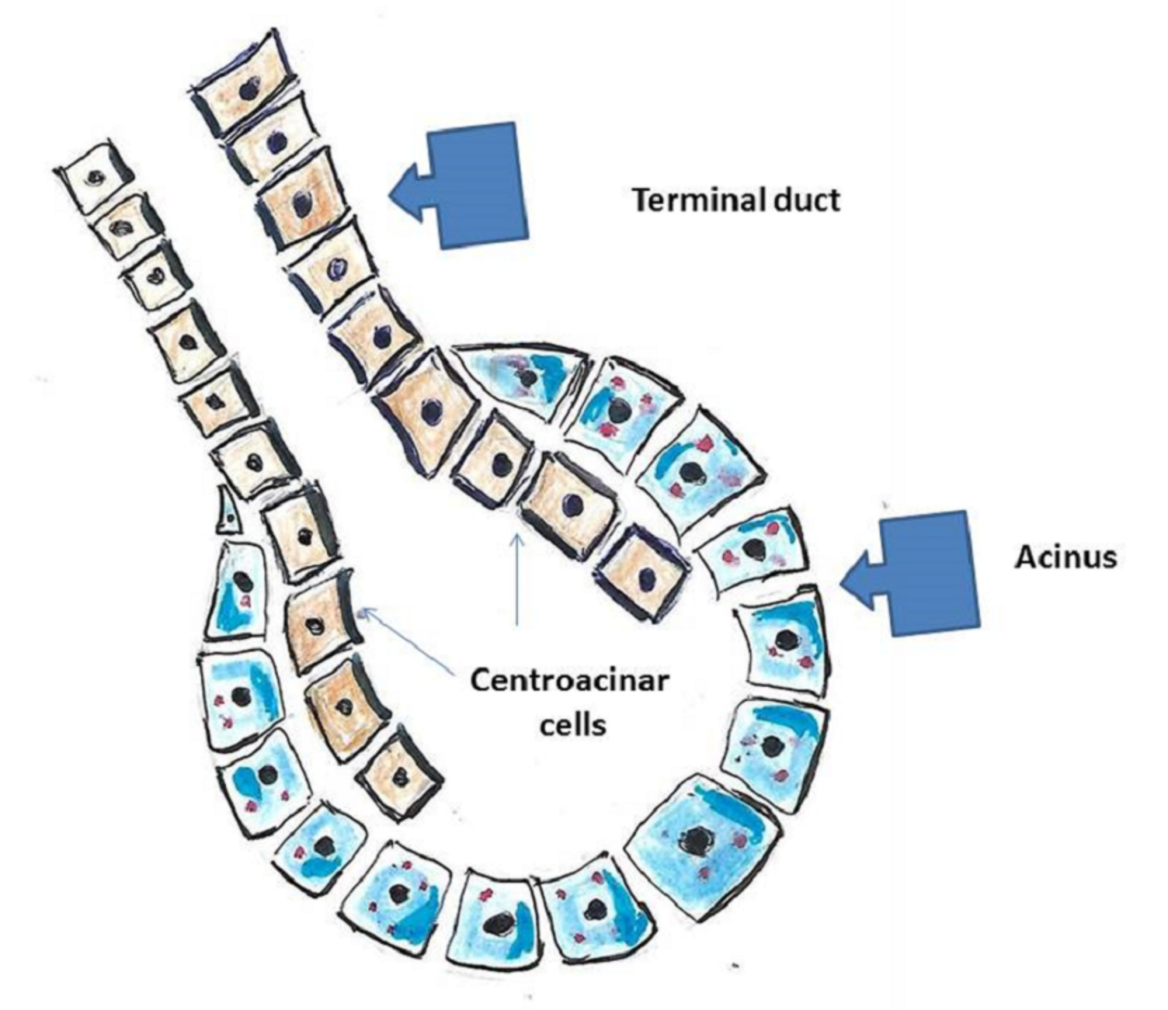
Exocrine glands

The exocrine gland classification is based on how the secretory cells produce their secretions (Figure 6).

Merocrine glands

**Figure 7:**
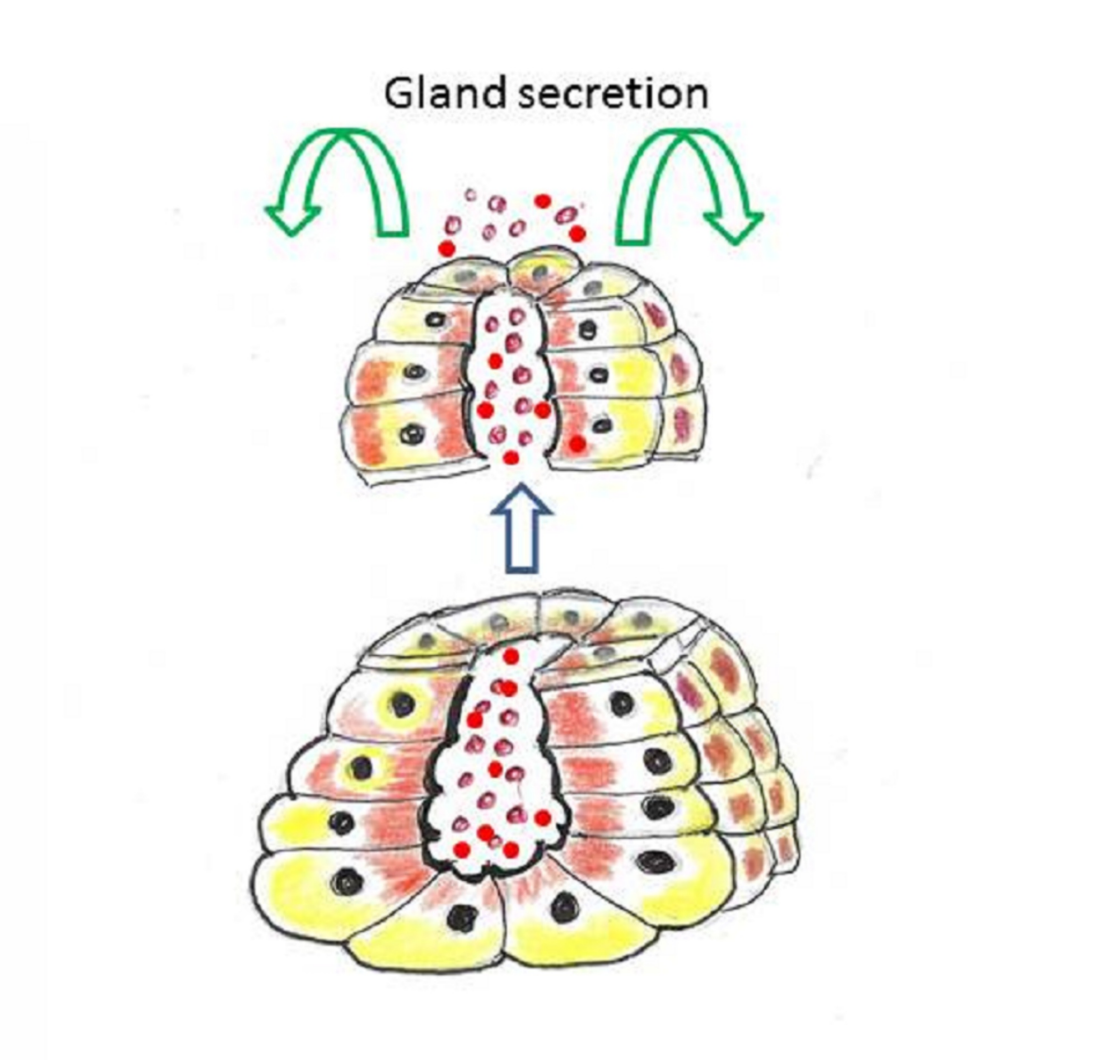
Merocrine glands

The merocrine (eccrine) gland secretions are excreted by exocytosis in the lumen of a duct system (Figure 7).

Holocrine glands

**Figure 8:**
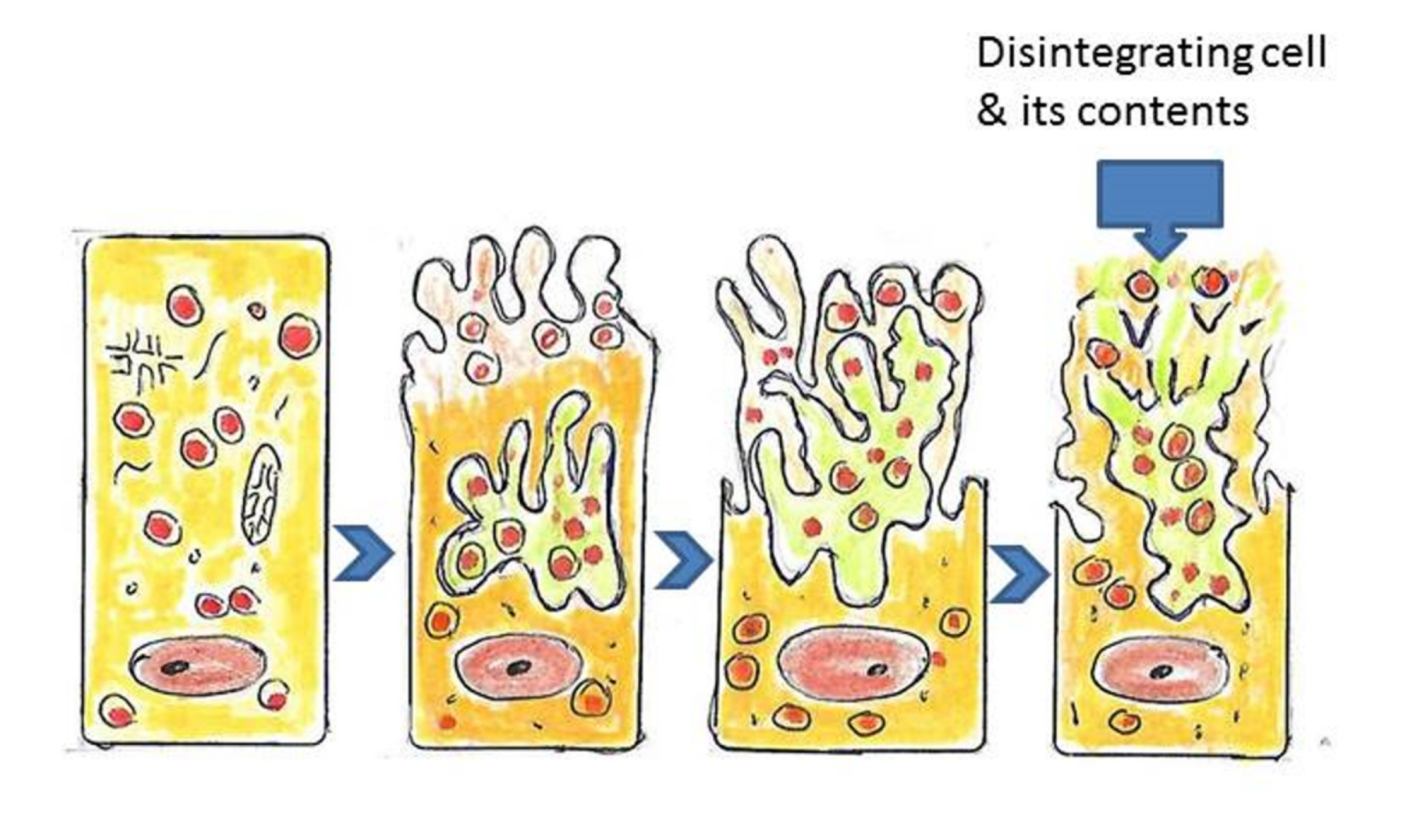
Holocrine cells

The holocrine gland secretions are initially produced in the cell cytoplasm, then the cell membrane ruptures, and the entire cell disintegrates to release its substance (Figure 8).

Apocrine glands

**Figure 9:**
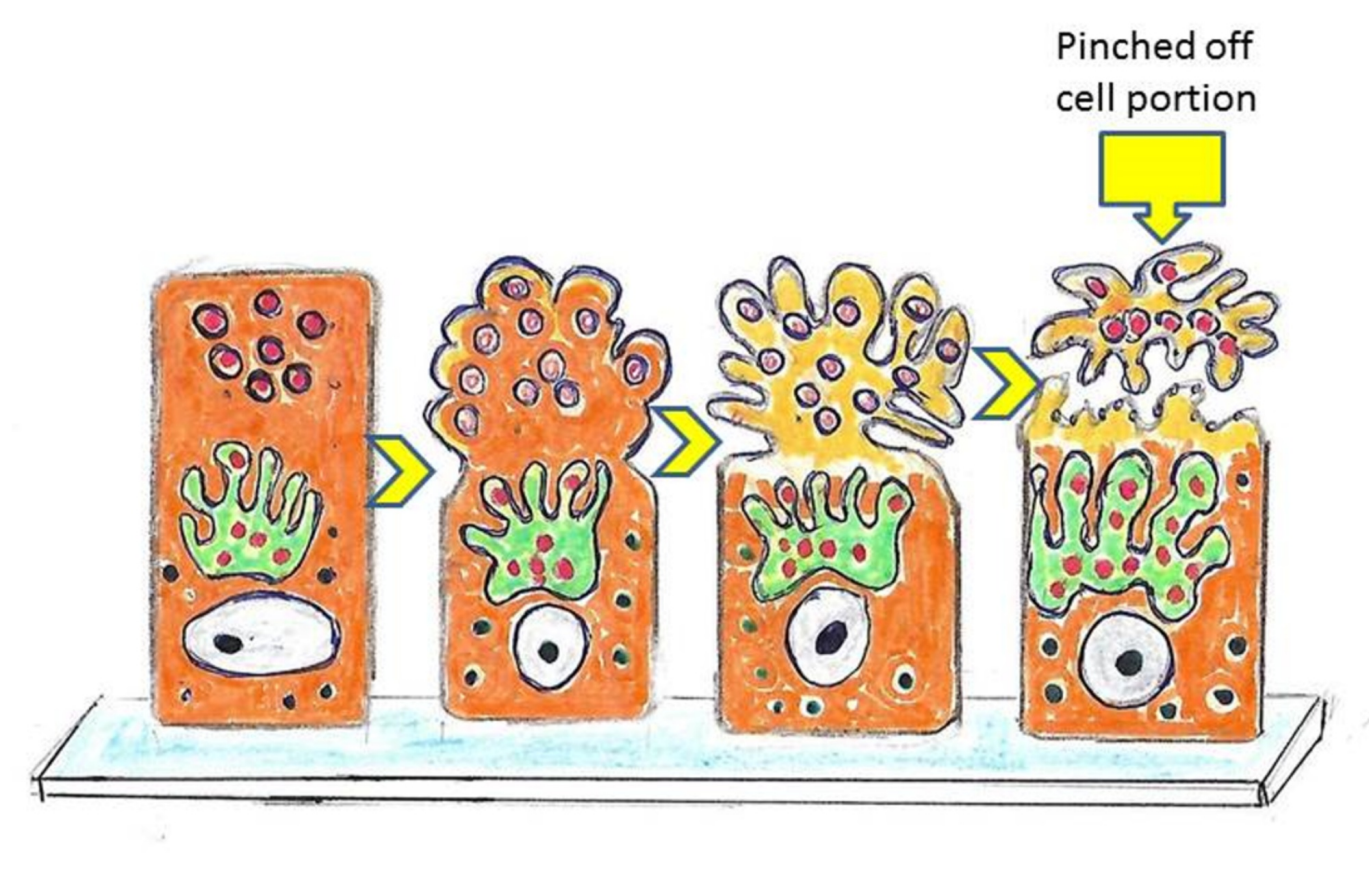
Apocrine cells

The apocrine gland cells bud their secretions off through the plasma membrane, producing extracellular membrane-bound vesicles (Figure 9).

Histologically, apocrine cells have an abundant eosinophilic or granular cytoplasm, and their nucleus contains distinct nucleoli in its round vesicular form [3].

Apocrine cells are divided into two categories. Type A apocrine cells have apical luminal blebbing or snouting and distinctive cell membranes. The apical portion of the cell contains coarse birefringent granules. The nuclei have globoid shape, often pale with one or two prominent nucleoli. A supranuclear iron-containing brown pigmented vacuole may be present [4].

Type B apocrine cells have a distinctly foamy cytoplasm, which contains small vacuoles that may coalesce and show lipofuscin pigment in their cytoplasm. The nuclei are usually located centrally and possess similar characteristics of type A apocrine cells [4].

The anatomical sites of the apocrine cells are axillary and groin sweat glands and the breast periareolar apocrine glands. They are also present in the perianal region, labia majora in women, and the scrotum and prepuce in men [5].

Histological characters of AAA

The term apocrine adenosis describes sclerosing adenosis present in the breast apocrine cells. However, in the presence of significant cytological atypical changes in the apocrine cells, such as thre-fold nuclear enlargement, prominent/multiple nucleoli, and hyperchromasia, the term apocrine atypia is used. When apocrine adenosis and apocrine atypia are superimposed, AAA is diagnosed [1]. AAA is a benign breast lesion and should not be regarded as a direct histologic precursor to breast carcinoma [6].

Radiological features

The mammogram may reveal the abnormality with or without amorphous or coarse heterogeneous calcifications. On ultrasonography, AAA may appear as irregular or circumscribed lesion. The magnetic resonance image may depict persistently enhancing focus [7].

Atypical apocrine metaplasia was not a common diagnosis in the past, and most of those diagnoses were based on findings in specimens. However, given current advancements in breast imaging techniques and the increased use of Tru-Cut® biopsy (Merit Medical, South Jordan, UT), the diagnosis of atypical apocrine metaplasia is encountered more often, even daily in large breast centres.

Immunohistochemistry

The absence of female sex hormone biomarkers (oestrogen and progesterone) and the presence of androgen receptor and GCDFP-15 confirm the diagnosis of apocrine adenosis with atypia [1]. Hematoxylin and eosin staining methods are mainly used for specimen study. It is important to have an accurate study of the specimens to avoid unnecessary surgical procedures.

GCDFP-15 is a glycoprotein originally isolated in the human breast. It is used as a specific immunohistochemical diagnostic marker for apocrine differentiation, especially in tumours originating in the breast (including apocrine carcinoma of the breast) [8-10].

Apocrine cells usually lack oestrogen and progesterone receptors, but they are positive for androgen receptors [1]. Our case specimen was negative for oestrogen receptors.

Cytokeratin (CK) 5 is a basic (type II) cytokeratin useful for detecting benign breast proliferation [11]. CK5 expression is variable in breast AAA [1]. It can also be detected in the basal-like subtype of invasive ductal carcinoma of the breast [10,12].

Transformation-related protein 63 (P63) is a protein encoded by the TP63 human gene. This marker is used for squamous differentiation, and its mutation is associated with Li-Fraumeni syndrome. It differentiates malignant conditions, which are often p53 positive, from reactive and metaplastic conditions, which are usually p53 negative [8,10]. P63 may show focal staining in AAA [2,13].

SMA is a marker found in stromal myofibroblastic cells, and it is used to identify myoepithelial cells in normal, neoplastic, or diseased breast [14,15].

Differential diagnosis and risk of malignancy

In 1990, Tavassoli and Norris suggested using the phrase atypical apocrine metaplasia, when a healthy breast gland is replaced by one layer of markedly pleomorphic apocrine cells with a threefold variation in nuclear size [16]. This is known as benign lesions; however, the risk of breast cancer is controversial. Breast AAA is of great importance to differentiate the histological picture of apocrine adenosis with atypia from cancerous and precancerous lesions as it may be misinterpreted as carcinoma. Breast AAA needs to be differentiated from low-grade apocrine DCIS; most of the low-grade apocrine DCIS have moderate pleomorphic nuclei and multiple nucleoli in addition to occasional large multinucleate cells [17].

Sometimes, breast pleomorphic lobular carcinoma in situ (PLCI) involves sclerosing adenosis. PLCI cells exhibit a significant degree of nuclear pleomorphism compared with classic lobular carcinoma in situ with prominent nucleoli, and their staining is positive for GCDFP-15 [18]. Breast AAA may be mistakenly diagnosed as breast invasive carcinoma due to the presence of enlarged cells and stromal distortion [1] (Table 1).

**Table 1:** Differential diagnosis of breast atypical apocrine adenosis DCIS, ductal carcinoma in situ

Differential Diagnosis
Breast atypical ductal hyperplasia
Breast pleomorphic lobular carcinoma in situ
Invasive ductal carcinoma
Low-grade apocrine DCIS
Breast invasive apocrine carcinoma

There is no direct link between AAA and malignancy, and the data from long-term follow-up studies of open biopsies suggest that AAA is not a high-risk or precursor lesion [19].

Fuehrer et al. from Mayo clinic published a cohort study in 2011, which included 37 women found to have apocrine adenosis with metaplasia in biopsy specimens [6]. Of the 37 patients, only three developed invasive ductal carcinoma or DCIS with a relative risk of 8% for cancer. This relative risk is similar to the general population relative risk for breast cancer. Hence, it was concluded that AAA is a benign, non-aggressive disease and does not progress into a malignant lesion.

Seidman et al. published observations about AAA and concluded that AAA confers an increased risk of developing breast carcinoma in women older than age 60 years, and the risk in younger women is probably low [20]. They advocated conservative management; however, the modality or the interval of follow-up was not specified.

## Conclusions

Apocrine change is becoming a frequent finding in breast specimens due to the increase in the number of the performed biopsies. Atypical apocrine features in sclerosing abnormality may be misdiagnosed as invasive carcinoma, and accurate diagnosis helps to avoid unnecessary overtreatment. While we believe it is a benign feature of the breast, further research is needed to set the guidelines for management and follow-up of patients with AAA.
